# Supplementary material for: Altered microRNA Transcriptome in Cultured Human Liver Cells upon Infection with Ebola Virus
Source: Int J Mol Sci. 2021 Apr 6;22(7):3792. doi: 10.3390/ijms22073792 (PMC8038836; doi:10.3390/ijms22073792)
Supplement: Supplementary file 1 [file ijms-22-03792-s001.zip › Supplementary_File/C_ GO_Analysis_Results/16-30nt_go_Reston-24h-Huh7_vs_Control-24h-Huh7_down.mature_mirna_targets/CC_Pvalue_tree.pdf]

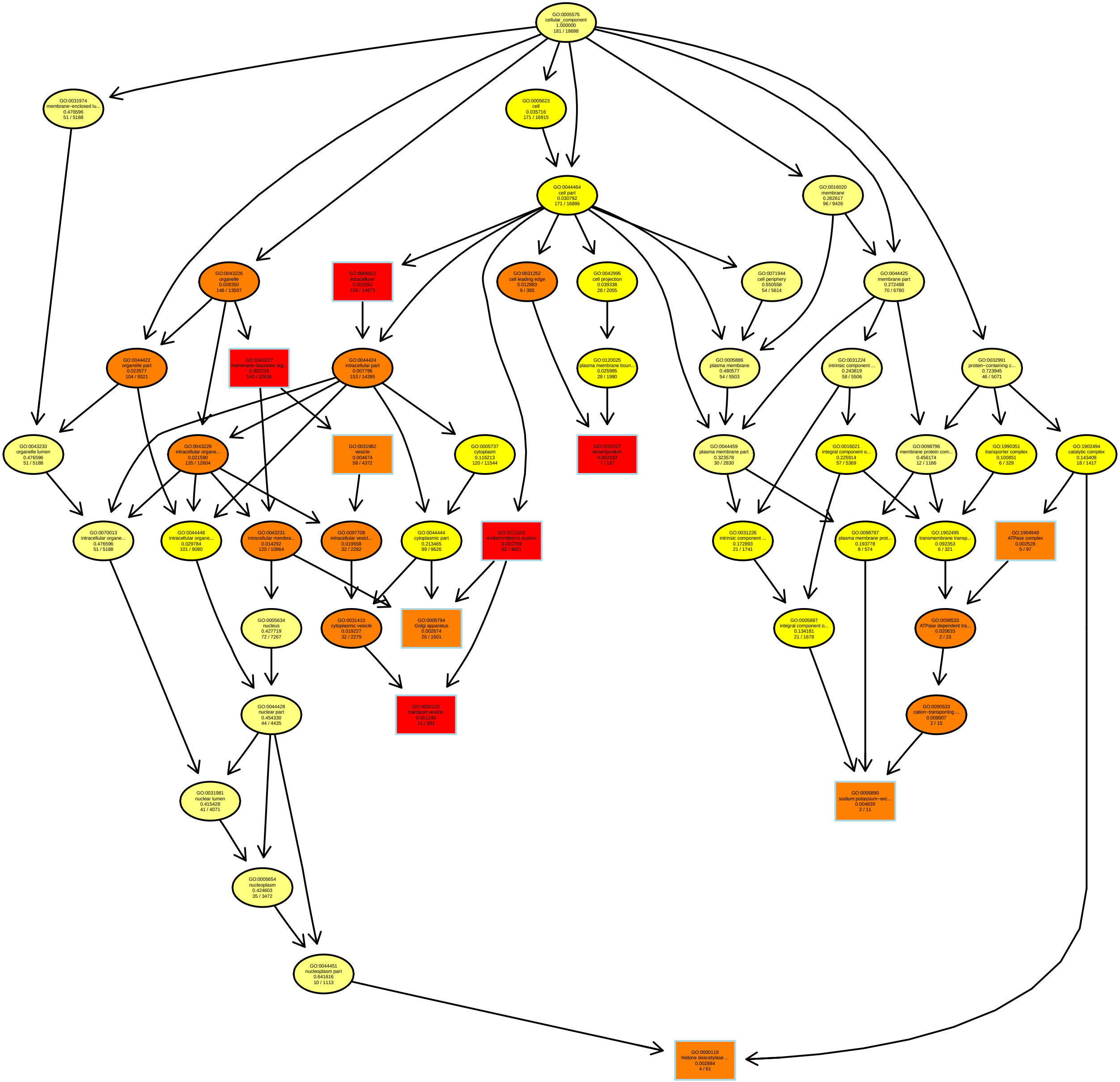
GO:0005575 cellular_component 1.000000 181 / 18698
GO:0031974 membrane−enclosed lu... 0.476596 51 / 5188
GO:0005623 cell 0.035716 171 / 16915
GO:0044464 cell part 0.030792 171 / 16886
GO:0016020 membrane 0.262617 96 / 9426
GO:0043226 organelle 0.008350 146 / 13597
GO:0005622 intracellular 0.001550 158 / 14673
GO:0031252 cell leading edge 0.012883 9 / 385
GO:0042995 cell projection 0.039338 28 / 2055
GO:0071944 cell periphery 0.550558 54 / 5614
GO:0044425 membrane part 0.272488 70 / 6780
GO:0044422 organelle part 0.023577 104 / 9321
GO:0043227 membrane−bounded org... 0.002216 140 / 12610
GO:0044424 intracellular part 0.007796 153 / 14395
GO:0120025 plasma membrane boun... 0.025985 28 / 1980
GO:0005886 plasma membrane 0.480577 54 / 5503
GO:0031224 intrinsic component ... 0.243819 58 / 5506
GO:0032991 protein−containing c... 0.723945 46 / 5071
GO:0043233 organelle lumen 0.476596 51 / 5188
GO:0043229 intracellular organe... 0.021590 135 / 12604
GO:0031982 vesicle 0.004674 58 / 4372
GO:0005737 cytoplasm 0.116213 120 / 11544
GO:0030027 lamellipodium 0.002333 7 / 187
GO:0044459 plasma membrane part 0.323578 30 / 2830
GO:0016021 integral component o... 0.225914 57 / 5369
GO:0098796 membrane protein com... 0.456174 12 / 1166
GO:1990351 transporter complex 0.100851 6 / 329
GO:1902494 catalytic complex 0.143409 18 / 1417
GO:0070013 intracellular organe... 0.476596 51 / 5188
GO:0044446 intracellular organe... 0.029784 101 / 9080
GO:0043231 intracellular membra... 0.014292 120 / 10864
GO:0097708 intracellular vesicl... 0.019558 32 / 2282
GO:0044444 cytoplasmic part 0.213465 99 / 9626
GO:0012505 endomembrane system 0.000769 62 / 4421
GO:0031226 intrinsic component ... 0.172893 21 / 1741
GO:0098797 plasma membrane prot... 0.193778 8 / 574
GO:1902495 transmembrane transp... 0.092353 6 / 321
GO:1904949 ATPase complex 0.002528 5 / 97
GO:0005634 nucleus 0.427719 72 / 7267
GO:0031410 cytoplasmic vesicle 0.019227 32 / 2279
GO:0005794 Golgi apparatus 0.002674 26 / 1501
GO:0005887 integral component o... 0.134181 21 / 1678
GO:0098533 ATPase dependent tra... 0.020633 2 / 23
GO:0044428 nuclear part 0.454330 44 / 4435
GO:0030133 transport vesicle 0.001249 11 / 381
GO:0090533 cation−transporting ... 0.009007 2 / 15
GO:0031981 nuclear lumen 0.415428 41 / 4071
GO:0005890 sodium:potassium−exc... 0.004839 2 / 11
GO:0005654 nucleoplasm 0.424603 35 / 3472
GO:0044451 nucleoplasm part 0.641616 10 / 1113
GO:0000118 histone deacetylase ... 0.002884 4 / 61
